# The effect of self-care counseling on health practices of adolescent pregnant women: a randomized controlled trial

**DOI:** 10.1186/s12884-021-04203-8

**Published:** 2021-10-27

**Authors:** Ronya Rezaie, Sakineh Mohammad-Alizadeh-Charandabi, Fatemeh Nemati, Mojgan Mirghafourvand

**Affiliations:** 1 Department of Midwifery, Faculty of Nursing and Midwifery, Tabriz University of Medical Sciences, Tabriz, Iran; 2 Faculty of Education and Psychology, University of Tabriz, Tabriz, Iran; 3 Social Determinants of Health Research Center, Faculty of Nursing and Midwifery, Tabriz University of Medical Sciences, Tabriz, Iran

## Abstract

**Background:**

Pregnancy and childbirth at an early age are associated with potential risks and complications for adolescent mothers. Health practices are behaviors that can positively affect maternal and fetal health. This study aimed to investigate the effects of self-care counseling on health practices (main outcome), attitudes towards motherhood and pregnancy, and pregnancy symptoms (secondary outcomes) in adolescent pregnant women.

**Methods:**

In this randomized controlled trial, 54 adolescent pregnant women admitted to the health centers of Bukan, Iran in 2020 were enrolled. Using randomized block design, the participants were randomly assigned to the counseling (*n*=27) and control (*n*=27) groups. Those in the intervention group attended 6 self-care group counseling sessions. The Health Practices Questionnaire-II (HPQ-II), Attitudes towards Motherhood and Pregnancy Questionnaire (PRE-MAMA), and Pregnancy Symptoms Inventory (PSI) were completed before and 4 weeks after the intervention.

**Results:**

Based on the results of ANCOVA with controlled baseline values, after the intervention the mean health practices score of the participants in the intervention group was significantly higher than those in the control group (adjusted mean difference (AMD): 36.34; 95% CI: 34.69 to 37.98; *P*<0.001). The mean attitude towards motherhood score of the participants in the intervention group was significantly higher than those in the control group (AMD: 1.01; 95% CI: 0.06 to 1.96; *P*= 0.038). However, the mean pregnancy symptoms score of the participants in the intervention group was partially lower than those in the control group (AMD: -1.37; 95% CI: -4.32 to 1.58; *P*= 0.354).

**Conclusion:**

Self-care counseling sessions can improve the health practices of adolescent pregnant women and enhance their attitudes towards maternal role and pregnancy. Therefore, planners are recommended to organize self-care counseling programs for all pregnant women, especially for adolescent pregnant women.

**Trial registration:**

Iranian Registry of Clinical Trials (IRCT): IRCT20120718010324N54. Date of registration: 2/3/2020. URL: https://en.irct.ir/user/trial/42571/view; Date of first registration: February 3, 2020.

## Background

Adolescence is a critical period and one of the most challenging stages of life [1]. It is a unique phase in the growth and development of women. According to the World Health Organization (WHO), individuals in the 10-19 years-old age group are considered as adolescent [2]. Adolescent pregnancy is a global issue. About 16 million girls in the 15-19 years-old age group and 2 million girls under the age of 15 get pregnant each year [3]. Adolescent pregnancy is associated with higher rates of morbidity and mortality of both the mother and infant [4]. They face problems such as inadequate nutrition and prenatal care, iron, iodine, folate and calcium deficiency, more risky behaviors like smoking and substance abuse, alcohol drinking and unsafe sexual activity. In addition, adolescent pregnant women have more psychological and social needs than older women [5].

Health practices are behaviors displayed by women during pregnancy that can affect maternal and fetal health and pregnancy outcomes [6]. Health practices that must be adopted during pregnancy include avoiding the use of cigarettes, alcohol, and other illicit substances, avoiding high-risk sexual behaviors, preventing the development of infections, avoiding over-the-counter medications, reducing caffeine intake, following a healthy diet, performing physical exercises regularly, getting adequate sleep and rest, taking necessary supplements (*e.g.* various vitamins, iron, and folic acid), maintaining oral hygiene, attending regular prenatal care and counseling sessions, and learning about pregnancy and childbirth [6–9]. Pregnant women are not often aware of the effect of healthy lifestyle on not only their health but also their children’s [10]. Some of these practices directly affect maternal and fetal or newborn outcomes (e.g., smoking), whereas others have indirect effects on outcomes through increasing the rate of pregnancy complications (e.g., excessive maternal weight gain). Some fetal or newborn outcomes that can be affected by these practices are low birth weight [11, 12], congenital anomalies [13], and spontaneous abortion [14]. Some long-term maternal outcomes include obesity [15], and, if poor health practices continue after birth, diabetes and heart disease may occur [16].

Pregnancy, as a period of adaptation to motherhood is a critical period and it brings a lot of changes for the woman [17]. There is an association between a woman’s antenatal psychological state and her adaptation for maternal functioning [18]. The attitudes of pregnant women towards motherhood play an important role influencing the ability of a woman to adapt to motherhood [17]. A negative attitude towards pregnancy and child care is correlated with later health problems of the child [18–20]. Adaptation abilities of women for motherhood can influence both their own wellbeing and health of the child [21]. Mothers who have positive attitudes towards their maternal role and believe that their health behaviors affect their infants’ health strive to improve their health and self-care practices to promote fetal health [6].

Women experience a series of changes during their pregnancy. Physical symptoms are common and are predominantly associated with normal physiological changes that occur during this time [22]. Healthy behaviors and other interventions during pregnancy have the potential to reduce the frequency and severity of annoying pregnancy symptoms, such as nausea, back pain, incontinence, quality of sleep, mood and libido [23]. Individual and group counseling services seem to improve health behaviors displayed by adolescent mothers [24].

Counseling is a professional relationship between a counselor and a client [25]. Health counseling can have many purposes [26]. During pregnancy, ongoing care is required to maintain and promote maternal and fetal health. Self-care is the first step towards improving maternal health [27]. Self-care refers to a set of deliberate, learned, and purposeful practices and activities performed by individuals to maintain and promote the health of themselves as well as their families, prevent development of diseases, and regain health after illness or injury [28]. In this process, individuals perform necessary actions by themselves, and health care providers merely provide them with necessary information and guidance [29]. Preconception health counseling has a positive effect on women’s health behaviors [30]. In addition, positive self-care practices during pregnancy can promote the health of mother and fetus, and reduce adverse outcomes of pregnancy [31, 32].

One of the main concerns of health care providers is to provide pregnant women with accurate information to make a positive change in their behavior [33]. Considering the high risk of adolescent pregnancy for maternal and neonatal health [34, 35], positive effects of health practices in reducing adverse maternal and neonatal outcomes [36], and the need to promote self-care practices in pregnant women, especially younger mothers, to ensure the health of themselves and their infants [31], counseling sessions must be held to promote the adoption of health practices by adolescent mothers.

The effect of self-care counseling on health practices has been assessed only in one study on 35 years of age or older pregnant women [37] and to our knowledge, there is no study in this regard in adolescent pregnant women. This study investigated the effects of self-care counseling on adoption of health practices (primary outcome), attitudes towards motherhood and pregnancy, and pregnancy symptoms (secondary outcomes) in adolescent pregnant women.

## Methods

### Study design and participants

In this randomized controlled trial, 54 adolescent pregnant women admitted to the health centers of Bukan, Iran were enrolled. Pregnant women in the 10-19 years-old age group and at the gestational age of 18-24 weeks who had at least middle school education, as well as a landline phone and a cellphone and were willing to participate in the study were enrolled. The exclusion criteria included having a high-risk pregnancy (due to diabetes, hypertension, cardiovascular diseases, lung diseases, *etc.*), suffering from a mental health problem, having a history of mental health problem or hospitalization in a psychiatric hospital, experiencing a disastrous life event in the past three months that can impair one’s mental health, and being prohibited to perform physical exercises.

The sample size was calculated based on the variable of health practices and using the results presented by Maddahi et al*.* [38]. It was considered as 23 per group with regard to the largest standard deviation of health practices, m_1_ = 123.57, m_2_ = 135.9, SD_1_ = SD_2_ = 11.14, α = 0.05, and Power = 95%. The final sample size was calculated as 27 by considering a loss to follow-up of 20%. Based on the results of this study, the trial has 99% actual power to detect a difference between groups.

### Sampling

The researcher selected socially and economically different health centers located in Bukan and visited these centers to obtain the list of mothers at the gestational age of 18-24 weeks using the Integrated Health System (IHS), known as the “SIB System”. The researcher called these mothers, provided them with a brief description of the research objectives and method, selected those meeting the inclusion criteria, and asked them to visit the relevant centers at a specific time if they were willing to participate in the study. In the first in-person meeting, the research objectives and method were fully explained to the women and those who were willing to participate were interviewed to complete the Health Practices in Pregnancy Questionnaire-II (HPQ-II). Those with moderate or lower HPQ-II scores were enrolled after completing and signing the informed consent forms. Other questionnaires were then completed by the participants.

### Random allocation

Allocation of participants to the study groups was random and each participant could not select the intervention group or control group. Using randomized block design (4 and 6-individual blocks), the participants were assigned to the intervention and control groups. To conceal the allocation sequence, the type of intervention was written on some papers which were placed in sealed envelopes. The sealed envelopes were then numbered and given to the participants based on their enrollment time. A person who not involved in the data collection and data analysis conducted the random allocation and allocation concealment.

### Intervention

The participants in the intervention group attended six 60-90 minute self-care group counseling sessions held by the researcher in a quiet and private room in a health center. The sessions were held at one week intervals and 5-7 individuals attended each session. The necessary information were provided to the mothers, and they were asked to share their personal opinions and experiences with others. In these sessions, the mothers learned about various health practices and their effects on maternal and fetal health and pregnancy outcomes. Table 1 indicates the contents of counseling sessions.

**Table 1:** Contents of the sessions

Sessions	Content	Homework
**Session 1**	Introduction of researcher and participant, specifying the session agenda, explanation the overall purpose of the research, description of mental and physical conditions by participant, explanations about the physiology and symptoms of pregnancy, handing them a booklet containing relevant educational contents	Reviewing the contents of session using the booklet
**Session 2**	Specifying the session agenda, reviewing previous session’s homework and answering participants’ questions, explanations about health practices in areas of learning about pregnancy and childbirth, importance of health and prenatal care	Reviewing the contents of session using the booklet
**Session 3**	Specifying the session agenda , reviewing previous session’s homework, explanations about health practices in areas of balance of rest and exercise and nutrition and diet care, expressing opinions and sharing experiences in the form of group discussions	Devise a 2-week diet and exercise plan
**Session 4**	Determining the session agenda ,reviewing previous session’s homework, explanations about the importance of preventing diseases and injuries, avoiding harmful drugs and narcotics	Reviewing the contents of session using the booklet
**Session 5**	Determining the session agenda and reviewing previous session’s homework, explanations about the importance of women’s attitude towards motherhood and pregnancy, relaxation activities (*e.g.* listening to a relaxing music, talking to and caressing the fetus, thinking about the baby, praying, *etc.*), breastfeeding benefits, and paying attention to their beauty and appearance	Reviewing the contents of session using the booklet
**Session 6**	Reviewing and summarizing the contents of all previous sessions, answering all questions raised by mothers	

The women in the control group only received routine pregnancy care provided by the health care providers. To observe ethical considerations, self-care booklets were also provided to the control group members at the end of the study.

### Data collection tools

The sociodemographic and obstetric questionnaire, Health Practices Questionnaire-II (HPQ-II), Attitudes towards Motherhood and Pregnancy Questionnaire (PRE-MAMA), and Pregnancy Symptoms Inventory (PSI) were completed by the researcher before and 4 weeks after the end of intervention using interview method.

The researcher-made sociodemographic and obstetric questionnaire included variables such as mother’s age, spouse’s age, educational qualifications, socio-economic status, *etc.* The qualitative face and content validity of this scale was confirmed.

The 34-item (HPQ-II) was developed in 2003 by Lindgren. It measures adequacy of health practices in six subdomains of balance of rest and exercise, disease and injury prevention, nutrition and diet care, avoidance of harmful drugs and narcotics, and obtaining knowledge about pregnancy and childbirth. The items are scored on a five-point Likert scale including “never” (score 1), “rarely” (score 2), “sometimes” (score 3), “often” score 4, and “always” (score 5) (Total score range: 34-170). Higher scores indicate that health practices are better adopted by mothers. The reliability of the Persian version of the tool was confirmed in a study in Sirjan-Iran (2014) with intra-class correlation coefficient (ICC) and Cronbach’s alpha coefficient of 0.81 and 0.83, respectively [9].

The Attitudes towards Motherhood and Pregnancy Questionnaire (PRE-MAMA) was designed by Ilska in 2014. This 11-item tool measures women’s attitudes towards their maternal role as well as their perceptions of child care and self-care. The items are scored on a four-point scale ranging from “not at all” (score 1) to “very much” (score 4) (Total score range: 11-44). The reliability of the scale has been confirmed (Cronbach’s alpha coefficient = 0.71) [39].

The Pregnancy Symptoms Inventory (PSI) has 41 items and measures the frequency and severity of pregnancy symptoms and their impacts on a pregnant mother’s life. Items related to the frequency of pregnancy symptoms are scored on a 4-point Likert scales including “never” (score 0), “rarely” (score 1), “sometimes” (score 2), and “often” (score 3). The pretest-posttest reliability of the tool for all items was between 0.5 and 1 (> 0.7 for 34 items) [23].

To assess the overall reliability of the HPQ-II, PRE-MAMA, and PSI, test-retest reliability and internal consistency of these tools were measured by calculating ICC and Cronbach’s alpha coefficient in a sample of 20 women at a two-week interval. ICC and Cronbach’s alpha coefficient of the HPQ-II, PRE-MAMA, and PSI were calculated as “0.97 and 0.83”, “0.97 and 0.73”, and “0.94 and 0.94” respectively.

### Data analysis

The data were analyzed in SPSS 24. The normality of the quantitative data was measured using the Kolmogorov-Smirnov (K-S) test. The independent t, chi-square, and Fisher’s exact tests were used to assess the homogeneity of the two groups in terms of sociodemographic and obstetric characteristics. The independent t-test and ANCOVA (with adjusting the baseline values) were used to compare the mean health practices, attitude towards motherhood and pregnancy, and pregnancy symptoms scores of the two groups before and after the intervention, respectively. The paired t-test was used to compare the pre-test and post-test scores within the groups. The results were analyzed using intention-to-treat analysis. *P*<0.05 was considered as significant.

## Results

**Fig. 1:**
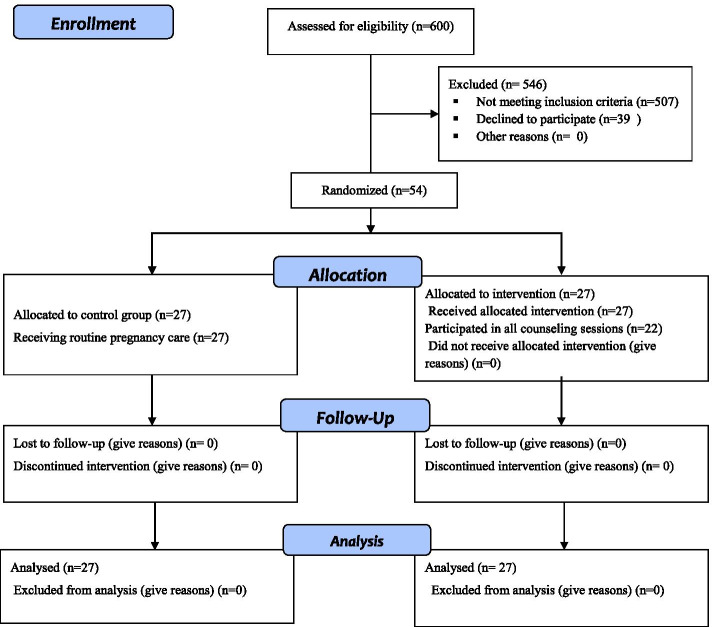
Study flow chart

Between July 2020 and February 2021, the researcher assessed 600 pregnant mothers, of whom 54 individuals were enrolled. The remaining 546 individuals were excluded due to their age (*n* = 400), high health practices score (*n* = 15), gestational age > 24 weeks (*n* = 60), physical problems (*n* = 12), poor educational qualifications (*n* = 20), and unwillingness to attend the counseling sessions (*n* = 39). Twenty two individuals attended all 6 counseling sessions. Three women failed to attend 2 counseling sessions due to their positive COVID-19 tests, and two women missed one session due to personal issues. No case of loss to follow-up was observed, and all 54 pregnant women were post-tested (Fig. 1).

The mean (SD) age of the participants in the intervention and control groups were 17.96 (1.05) and 18.07 (0.96), respectively. The mean (SD) gestational age of the participants in the intervention and control groups were 20.89 (1.94) and 20.77 (1.67), respectively. About half of the women in the intervention group (48.1%) and one third of those in the control group (33.3%) had a high school diploma. Most of the women in both the intervention and control groups (85.3%) were housewives. The majority of the participants in both the intervention (74.1%) and control groups (85.2%) experienced their first pregnancy. More than half of the women in both the intervention (63%) and control groups (51.9%) were having a planned pregnancy. The majority of the participants in the intervention (85.2%) and control groups (88.9%) were satisfied with the gender of their infants (Table 2).

**Table 2:** The sociodemographic and obstetric characteristics of participants

Variable	Counselling group (***n***=27)	Control group (***n***=27)
	Mean ( Std. Deviation)	Mean ( Std. Deviation)
**Age (years)**	17.96 (1.05)	18.07 (0.96)
**Husband’s age** (Year)	25.29 (3.46)	25.78 (3.14)
**Marriage age** (Year)	16.96 (1.25)	16.78 (0.93)
**Start of sexual activity with husband** (Year)	16.96 (1.25)	16.85 (0.86)
**Menarche age** (Year)	12.15 (0.95)	12.33 (0.96)
**Gestational age** (Weeks)	20.89 (1.95)	20.78 (1.67)
**Body Mass Index** (Kg/m^2^)	24.29 (3.76)	25.20 (3.91)
	**Number (percent)**	**Number (percent)**
**Home status**		
Private home	9 (33.3)	11 (40.7)
Rental home	7 (25.9 )	8 (29.6)
Relative’s home	11 (40.7)	8 (29.6)
**Live with**		
Your family	1 (3.7)	1 (3.7)
Husband’s family	11 (40.7)	11 (40.7)
Only Husband	15 (55.6)	15 (55.6)
**Nutrition with family**		
Yes	11 (40.7)	11 (40.7)
No	16 (59.3)	16 (59.3)
**Income status**		
Full sufficient	6 (22.2)	4 (14.8)
Almost sufficient	18 (66.7)	20 (74.1)
Not sufficient at all	3 (11.1)	3 (11.1)
**Mother’s education**		
Middle school	7 (25.9 )	9 (33.3)
High school	13 (48.1)	9 (33.3)
Diploma	6 (22.2)	9 (33.3)
University	1 (3.7)	0 (0.0)
**Husband’s education**		
Elementary	1 (3.7)	1 (3.7)
Middle school	0 (0.0)	6 (22.2)
High school	9 (33.3)	8 (29.6)
Diploma	15 (55.6)	7 (25.9)
University	2 (7.4)	5 (18.5)
**Mother’s job**		
House wife	23 (85.2)	23 (85.2)
Employed	1 (3.7)	0 (0.0)
Student	3 (11.1)	4 (14.8)
**Husband’s job**		
Unemployed	2 (7.4)	0 (0.0)
Employee	0 (0.0)	2 (7.4)
Worker	11 (40.7)	9 (33.3)
Self employed	14 (51.9)	16 (59.3)
**Husband’s support**		
Very much	5 (18.5)	10 (37.0)
Much	17 (63.0)	10 (37.0)
Moderate	5 (18.5)	5 (18.5)
Low	0 (0.0)	2 (7.4)
**Marital satisfaction**		
Very much	8 (29.6)	6 (22.2)
Much	10 (37.0)	13 (48.1)
Moderate	9 (33.3)	8 (29.6)
**Gravida**		
First	20 (74.1)	23 (85.2)
Second	7 (25.9 )	4 (14.8)
**Living child**		
Zero	26 (96.3)	26 (96.3)
One	1 (3.7%)	1 (3.7)
**Abortion**		
Zero	21 (77.8%)	24 (88.9%)
One	6 (22.2%)	3 (11.1%)
**Prenatal care status**		
Regularly	21 (77.8%)	16 (59.3%)
Irregularly	6 (22.2%)	11 (40.7%)
**Pregnancy status**		
Planned pregnancy	17 (63.0%)	14 (51.9%)
Unplanned pregnancy	10 (37.0%)	13 (48.1%)
**Mother’s satisfaction of child gender**		
Satisfied	23 (85.2)	24 (88.9)
Unsatisfied	4 (14.8)	3 (11.1)
**Father’s satisfaction of child gender**		
Satisfied	26 (96.3)	25 (92.6)
Unsatisfied	1 (3.7)	2 (7.4)

Before the intervention, the mean (SD) health practices score in the intervention and control groups was 91.85 (5.83) and 92.55 (3.88), respectively. The independent t-test results showed no significant difference between the two groups before the intervention (*P* = 0.604). After the intervention, the mean (SD) score of health practices in the intervention and control groups was 129.74 (12.5) and 93.85 (3.37), respectively. The results of ANCOVA (with controlled baseline values) showed that the mean health practices score of women in the intervention group was significantly higher than those in the control group (MD: 36.34; 95% CI: 34.69 to 37.98; *P*< 0.001). Also, there was significant difference in the results from pretest to post-test within the intervention (*P*<0.001) and the control (*P*= 0.015) groups. In addition, there were no significant differences between the two groups of study at pretest in terms of all subdomains of health practices. However, after the intervention, there were significant differences between the two groups in terms of all subdomains of health practices (Table 3).

**Table 3:** Comparison of total health practices score and its subscales in the study groups

Variable	Counseling group (***n***=27) Mean (SD^⁕^)	Control group (***n***=27) Mean (SD^⁕^)	Mean difference (95% confidence interval)	***P***-value
**Total health practice score** (Score range: 34-170)				
Before intervention	91.85 (5.83)	92.55 (3.88)	-0.70 (-3.41 to 2.00)	0.604^‡^
After intervention	129.74 (12.5)	93.85 (3.37)	36.34 (34.69 to 37.98)	<0.001^†^
Pre- vs post- intervention (*P*-value)^§^	<0.001	0.015		
**Health practices subscales**				
**Balance between rest and exercise** (Score range: 4-20)				
Before intervention	5.52 (0.97)	5.37 (1.08)	0.15 (-0.41 to 0.71)	0.599^‡^
After intervention	9.67 (1.18)	4.81 (0.92)	4.78 (4.26 to 5.30)	<0.001^†^
Pre- vs post- intervention (*P*-value)^§^	<0.001	0.002		
**Diseases and injury prevention** (Score range: 5-25)				
Before intervention	14.78 (1.98)	15.23 (1.69)	-0.55 (-1.56 to 0.45)	0.273^‡^
After intervention	22.18 (1.07)	15.89 (1.39)	6.54 (6.02 to 7.07)	<0.001^†^
Pre- vs post- intervention (P-value)^§^	<0.001	0.045		
**Nutrition and diet care** (Score range: 7-35)				
Before intervention	22.26 (2.31)	22.44 (2.41)	-0.18 (-1.47 to 1.10)	0.774^‡^
After intervention	36.00 (2.15)	22.59 (1.91)	13.49 (12.53 to 14.44)	<0.001^†^
Pre- vs post- intervention (*P*-value)^§^	<0.001	0.602		
**Obtaining health care** (Score range: 7-35)				
Before intervention	13.15 (1.75)	13.59 (1.47)	-0.44 (-1.33 to 0.44)	0.317^‡^
After intervention	18.00 (1.57)	13.22 (1.60)	5.04 (4.34 to 5.71)	<0.001^†^
Pre- vs post- intervention (*P*-value)^§^	<0.001	0.076		
**Obtaining knowledge about pregnancy and childbirth** (Score range: 5-25)				
Before intervention	16.15 (2.44)	15.81 (2.52)	0.33 (-0.78 to 1.44)	0.550^‡^
After intervention	23.89 (1.89)	17.33 (1.41)	6.37 (5.69 to 7.05)	<0.001^†^
Pre- vs post- intervention (*P*-value)^§^	<0.001	<0.001		

^⁕^ Standard Deviation

^‡^ Independent t-test

^†^ANCOVA

^§^Paired t-test

Before the intervention, the mean (SD) attitude towards motherhood and pregnancy score in the intervention and control groups was 32.26 (4.57) and 31.48 (3.98), respectively. The independent t-test results indicated no significant difference between the two groups before the intervention (*P* = 0.508). After the intervention, the mean (SD) score of health practices in the intervention and control groups was 33.92 (3.16) and 32.44 (3.08), respectively. The ANCOVA results showed that the mean attitude towards motherhood and pregnancy score of women in the intervention group was significantly higher than those in the control group (MD: 1.01; 95% CI: 0.06 to 1.96; *P* = 0.038). Also, there was significant difference in the results from pretest to post-test within the intervention (*P*= 0.005) and the control (*P*= 0.016) groups (Table 4).

**Table 4:** Comparison of attitudes towards motherhood and pregnancy and pregnancy symptoms scores in the study groups

Variable	Counseling group (***n***=27) Mean (SD^**⁕**^)	Control group (***n***=27) Mean (SD^**⁕**^)	Mean difference (95% Confidence interval)	P-value
**Attitude toward motherhood and pregnancy** (Score range: 11-44)				
Before intervention	32.26 (4.57)	31.48 (3.98)	0.78 (-1.56 to 3.12)	0.508^‡^
After intervention	33.92 (3.16)	32.44 (3.08)	1.01 (0.06 to 1.96)	0.038^†^
Pre- vs post- intervention (P-value)^§^	0.005	0.016		
**Pregnancy symptoms** (Score range: 0-123)				
Before intervention	30.78 (10.54)	29.55 (7.98)	1.22 ( -3.89 to 6.33)	0.633^‡^
After intervention	25.92 (6.51)	27.15 (4.14)	-1.37 (-4.32 to 1.58)	0.354^†^
Pre- vs post- intervention (P-value)^§^	<0.001	0.129		

^⁕^ Standard Deviation

^‡^ Independent t-test

^†^ANCOVA

^§^Paired t-test

Before the intervention, the mean (SD) attitude towards motherhood and pregnancy score of the participants in the intervention and control groups was 30.78 (10.54) and 29.55 (7.98), respectively. The independent t-test results indicated no significant difference between the two groups before the intervention (*P*= 0.633). After the intervention, the mean (SD) pregnancy symptoms score of the participants in the intervention and control groups was 25.92 (6.51) and 27.15 (4.14), respectively. Based on the ANCOVA results, the mean pregnancy symptoms score of the participants in the intervention group was partially lower than those in the control group (MD: - 1.37; 95% CI: - 4.32 to 1.58; *P* = 0.354). There was significant difference in the results from pretest to post-test within the intervention (*P*<0.001) and no change in the results within the control group (*P*= 0.129) (Table 4).

## Discussion

The results indicated that self-care counseling positively affects health practices and its subdomains, attitudes towards motherhood and pregnancy.

After the intervention, the overall health practices score of women in the intervention group was significantly higher than those in the control group. Accordingly, other studies have shown that counseling improves health practices. For example, Mohaddesi et al. investigated the effect of counseling on health-promoting behavior of mothers with gestational diabetes. Based on their findings, counseling significantly increased the emergence of health-promoting behaviors in the intervention group members compared with those in the control group [40]. In their study entitled “Associations between preconception counseling and maternal behaviors before and during pregnancy”, Williams et al. found that preconception counseling is associated with positive maternal behaviors such as regular consumption of necessary supplements, regular prenatal care, cessation of smoking, drinking cessation. These behaviors increase the likelihood of a healthy mother, infant, and pregnancy [30]. Aghababaei et al. investigated the effect of self-care counseling on health practices in 35 years of age or older pregnant women and concluded that the intervention has led to a significant increase in the mean health practices score of women in the intervention group [37]. Jaras et al. compared the effectiveness of focus group discussion and teach-back self-care training on the lifestyle of pregnant women. The intervention significantly increased all aspects of lifestyle (except stress management) in the two intervention groups [41]. Health practices play a prominent role in reducing pregnancy and childbirth complications in adolescents by reducing relevant risk factors [42]; therefore, the results indicate that self-care counseling can probably reduce the adverse outcomes of pregnancy and childbirth in adolescents [43].

Based on the present results, counseling significantly improved the participants’ attitudes towards motherhood and pregnancy. No study was found to investigate the effect of counseling on mothers’ attitudes towards pregnancy or their motherhood role; therefore, the researcher compared the results with the findings of studies that have examined the effect of counseling and training on mothers’ attitudes toward other issues. For example, Masoumi et al. investigated the effect of exclusive breastfeeding counseling on awareness and attitudes of mothers. The results showed that counseling increases awareness and attitudes of mothers in the intervention group compared with those in the control group [44]. Panahi et al. found that training of parents improves their knowledge and attitude and enhances mothers’ exclusive breastfeeding performance [45]. In line with the above finings, Khodakarami et al. examined the effect of group counseling on attitudes of women who consider children as the pillar of life. Their findings revealed some improvements in the attitudes of the participants [46]. Women who cannot properly prepare themselves for their new role, as a mother, will experience many problems even after the childbirth [47]; therefore, some measures must be taken to improve the attitudes of such women towards their maternal role.

Despite the partial decrease in pregnancy symptoms experienced by women in the intervention group compared with those in the control group, no significant difference was observed between the two groups. Some studies have investigated the effect of counseling on some symptoms of pregnancy such as nausea and vomiting. Emami Sahebi et al. (2017) enrolled 50 pregnant women with moderate to severe nausea and vomiting to examine the effect of behavioral therapy on nausea and vomiting of pregnancy. Women in the intervention group attended 6 individual behavioral therapy sessions. Based on the results, the frequency of vomiting in the intervention group was 7.2% lower than in the control group after the intervention [48]. Sangestani et al. conducted a study entitled “Effect of family centered consultation on nausea and vomiting in pregnancy: a clinical trial study”. They observed significant reductions in nausea and vomiting scores of mothers in the intervention group compared with those in the control group [49]. Pregnancy symptoms and problems such as varicose veins, limb swelling, fatigue, and lower back pain are less common in physically active women [50]. These women are also less likely to experience sleep disorders, respiratory problems, stress, and depression, and can easily adapt to physiological changes during pregnancy [51, 52]. Health practices and lifestyles adopted by pregnant women generally affect the frequency and severity of pregnancy symptoms such as nausea, lower back pain, urinary incontinence, sleep quality, mental state and especially mood [23]. Therefore, self-care counseling seems to reduce pregnancy symptoms experienced by pregnant women through improving their health practices.

Considering the high risk of adolescent pregnancy and adverse effects of poor health practices on maternal and fetal health, based on the result of this study it can be stated that self-care counseling improves health practices adopted by this group of mothers. Therefore, health care providers can use this approach to maintain and improve the health of adolescent pregnant women and reduce the adverse outcomes of pregnancy.

### Strengths and limitations

In this study, all principles of clinical trial design such as random allocation and allocation concealment were observed. The psychometric properties of the standardized questionnaires used in this study have been confirmed in the studies carried out in Iran. The intervention was designed with regard to the cultural and moral values of the target population. Finally, no case of loss to follow-up was observed in the present study.

This study merely focused on adolescent pregnant women who had a poor performance in adopting health practices. Given the fact that most adolescent mothers come from middle and poor classes with a low socioeconomic status (*i.e.* the low rate of adolescent marriage and pregnancy among families with a high socioeconomic status), the findings cannot be generalized to all pregnant women.

## Conclusion

Group counseling sessions can improve the health practices of adolescent pregnant women and enhance their attitudes towards maternal role and pregnancy. Adolescence is a very important period of a life and pregnancy is also a critical life event; therefore, adoption of poor health practices during this period can have irreversible effects on the health of mother and child. Considering the high risk of adolescent pregnancy and relevant maternal and neonatal complications, planners are recommended to organize self-care counseling programs for adolescent pregnant women in all health centers.

## Data Availability

The datasets used and/or analysed during the current study available from the corresponding author on reasonable request.

## Abbreviations

HPQ-II: Health Practices Questionnaire-II
PRE-MAMA: Attitudes towards Motherhood and Pregnancy Questionnaire
PSI: Pregnancy Symptoms Inventory
ANCOVA: Analysis of Covariance
MD: Mean Difference
95% CI: 95% Confidence Interval
WHO: World Health Organization
HIS: Integrated Health System
K-S: Kolmogorov-Smirnov
